# Antibody switch AIDed by pyruvate

**DOI:** 10.1093/lifemeta/loae001

**Published:** 2024-01-05

**Authors:** Haoming Luan, Tiffany Horng

**Affiliations:** School of Life Sciences and Technology, ShanghaiTech University, Shanghai 201210, China; School of Life Sciences and Technology, ShanghaiTech University, Shanghai 201210, China

**Metabolism is mobilized to meet the biosynthetic, bioenergetic, and regulatory demands of immune cell activation. Almost nothing is known about the metabolic basis of antibody class switch in B cells. A recent study published in *Nature Communications* through a joint effort by Chen Lab (metabolism biology) and Liu Lab (B cell biology) from Tsinghua University reveals that the monocarboxylate carrier MCT1 (monocarboxylate transporter 1)** **promotes pyruvate oxidation and histone acetylation to support class switch recombination (CSR), the process by which B cells make antibodies of different isotypes with unique effector functions.**

Antibody memory is the basis for nearly all successful vaccines. B lymphocytes are responsible for the protective antibody responses arising from the recognition of the pathogen-associated antigens by B cell receptors (BCRs). During the first encounter with antigen, the IgM-BCRs expressing naïve B cells generate low-titered primary antibody responses, while in response to antigen recall, memory B cells that express class-switched IgG-BCRs are mainly responsible for the memory antibody response and high-titered IgG antibody responses. This is fundamentally different from the strategy used by all other immune cells (including T cells) that lack class switch recombination (CSR). Despite the rapid rise of the immunometabolism field in recent years, how cellular metabolism supports the functions of B cells, especially differentiated B cells like germinal center (GC) B cells (GCBCs) and plasma cells, has significantly lagged behind that of other activated immune cells like T cells and macrophages. Thus there is an unmet basic need to investigate the metabolic basis of CSR in B cells. For example, aerobic glycolysis is critical for rapidly proliferating cells [1] and presumably also for the rapidly dividing GCBCs, but while some studies indicate the enhanced dependence of GCBCs on glucose utilization, other studies instead point to a requirement for fatty acid oxidation [2, 3]. Clearly much remains unknown regarding the metabolic basis of B cell activation and differentiation.

Monocarboxylate transporters (MCTs) transport monocarboxylates, and MCT1, whose most important substrates include lactate and pyruvate, is known to be important in supporting aerobic glycolysis in rapidly proliferating cells like tumor cells and activated CD8 T cells [4]. However, the role of MCTs in B cell activation and differentiation remains elusive. In this paper [5], the authors started their study by comparing the expression of various MCTs in activated B cells and found that MCT1 was expressed at the highest level. Using newly generated mice in which MCT1 is specifically deleted in the B cell lineage, the authors found that B cell development in the bone marrow and B cell homeostasis in the periphery were unimpaired. However, upon challenging with a T cell-dependent model antigen, the authors found that MCT1-deficient B cells mounted a normal antigen-specific IgM response, but produced antigen-specific IgG that was of lower quantity and affinity. GCBC differentiation and proliferation were also reduced in the absence of MCT1. Together, these findings indicated a defect of CSR in MCT1-deficient B cells.

**Figure 1:**
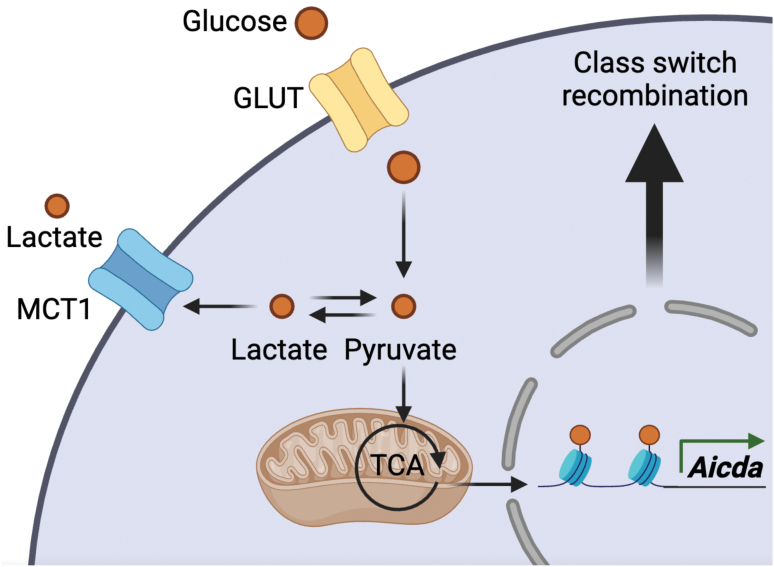
In GCBCs, glucose acts as a metabolic substrate for histone acetylation to support the expression of genes including *Aicda*, which regulates CSR and affinity maturation

MCT1 substrates lactate and pyruvate are intermediates in aerobic glycolysis, prompting the authors to examine glucose metabolism. Metabolic analyses indicated that activated MCT1-deficient B cells downregulated aerobic glycolysis, but upregulated pyruvate oxidation and oxidative metabolism, relative to wild-type (WT) counterparts. Presumably, the absence of MCT1 forced a shunting of pyruvate toward oxidation in the tricarboxylic acid (TCA) cycle. By tracing glucose and pyruvate into glycolysis, TCA cycle, and ultimately into histones, the authors nicely showed that in WT-activated B cells, pyruvate acted as a metabolic substrate for histone acetylation to support a global increase in histone acetylation. Such increase in histone acetylation drove enhanced expression of *Aicda*, the gene encoding activation-induced cytidine deaminase (AID), which mediates CSR and affinity maturation (Fig. 1). The precise mechanism of pyruvate redistribution between the nuclear and non-nuclear compartments, to be explored in future studies, is presumably important for epigenetic modifications and energy production. These findings are also reminiscent of other studies showing that glucose/pyruvate oxidation in activated macrophages and T cells can influence histone acetylation and gene expression to promote the activated phenotype [6, 7]. Intriguingly, relative to WT counterparts, activated MCT1-deficient B cells had reduced histone acetylation at the *Aicda* promoter, despite enhanced pyruvate oxidation. Although the mechanistic basis remained unclear, the findings nevertheless implicated dysregulated pyruvate metabolism and histone acetylation at *Aicda* as the basis of attenuated CSR in MCT1-deficient B cells.

Finally, the study addressed the disease relevance of their basic findings in a genetically modified murine model. Although B cells are protective against pathogens, they contribute to disease pathogenesis in autoimmune diseases like systemic lupus erythematosus (SLE), where autoimmune antibodies are known to be positively correlated with the clinical manifestations of SLE. First, by analyzing both existing and newly established RNA-seq datasets from multiple sources, the authors found that MCT1 levels were increased in switched memory B cells from SLE patients, relative to healthy controls. Next, the authors showed that mice with MCT1-deficient B cells were protected in a mouse model of SLE, displaying reduced splenomegaly, numbers of GCBCs and IgG1^+^ B cells, and titers of autoreactive, anti-dsDNA (double-stranded DNA) antibodies. Finally, administration of an MCT1 inhibitor in this model reduced titers of anti-dsDNA antibodies. Intriguingly, the authors also revealed that a clinically approved SLE drug, dexamethasone, was particularly effective in decreasing the expression levels of MCT1 from SLE patients *in vitro*. In light of these findings, future studies to further investigate biological mechanisms are warranted.

In summary, this comprehensive study uncovered an important role for MCT1 in regulating pyruvate metabolism to modulate AID expression and CSR in activated B cells. The findings have relevance for our basic understanding of how metabolism influences humoral immunity in settings of health and disease and may pave the way toward opening new therapeutic windows in SLE.
